# Biological mesh is a safe and effective method of abdominal wall reconstruction in cytoreductive surgery for peritoneal malignancy

**DOI:** 10.1002/bjs5.93

**Published:** 2018-08-02

**Authors:** A. Tzivanakis, S. P. Dayal, S. J. Arnold, F. Mohamed, T. D. Cecil, A. K. Venkatasubramaniam, B. J. Moran

**Affiliations:** ^1^ Peritoneal Malignancy Institute Basingstoke and North Hampshire Hospital Basingstoke RG24 9NN UK

## Abstract

**Background:**

Patients with peritoneal malignancy often have multiple laparotomies before referral for cytoreductive surgery (CRS). Some have substantial abdominal wall herniation and tumour infiltration of abdominal incisions. CRS involves complete macroscopic tumour removal and hyperthermic intraperitoneal chemotherapy (HIPEC). Abdominal wall reconstruction is problematic in these patients. The aim of this study was to establish immediate and long‐term outcomes of abdominal wall reconstruction with biological mesh in a single centre.

**Methods:**

A dedicated peritoneal malignancy database was searched for all patients who had biological mesh abdominal wall reconstruction between 2004 and 2015. Short‐ and long‐term outcomes were reviewed. All patients had annual abdominal CT as routine peritoneal malignancy follow‐up.

**Results:**

Some 33 patients (22 women) with a mean age of 53·4 (range 19–82) years underwent abdominal wall reconstruction with biological mesh. The majority (23) had CRS for pseudomyxoma (19 low grade), six for colorectal peritoneal metastasis and four for appendiceal adenocarcinoma; 18 had undergone CRS and HIPEC previously. Twenty‐five of the 33 patients had abdominal wall tumour involvement and eight had concurrent hernias. The mean duration of surgery was 486 (range 120–795) min and the mean mesh size used was 345 (50–654) cm^2^. Ten patients developed wound infections and four had a seroma. Two developed early enterocutaneous fistulas. Mean follow‐up was 48 months. Five patients developed an incisional hernia. Four died from progressive malignancy. A further 15 patients had disease recurrence, but only one had isolated abdominal wall recurrence.

**Conclusion:**

Biological mesh was safe and effective for abdominal wall reconstruction in peritoneal malignancy. Postoperative wound infections were frequent but nevertheless incisional hernia rates were low with no instances of mesh‐related bowel erosion or fistulation.

## Introduction

**Figure 1:**
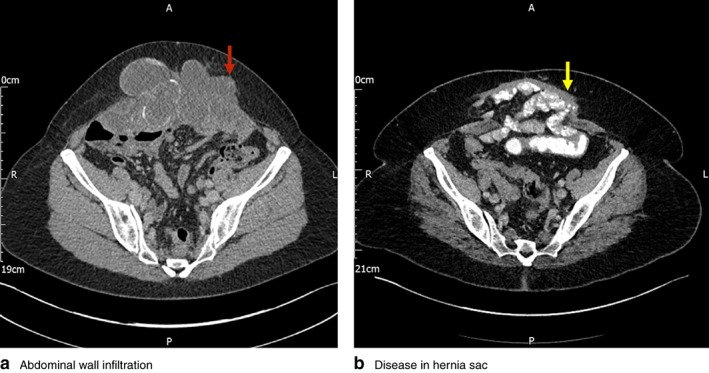
CT images demonstrating **a** abdominal wall infiltration by tumour (red arrow) and **b** incisional hernia with disease within hernia sac (yellow arrow)

**Figure 2:**
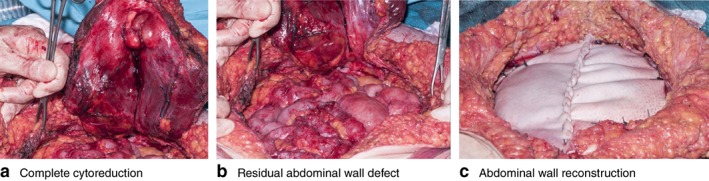
Extensive abdominal wall excision with reconstruction using biological mesh: **a** abdominal wall excision to achieve complete cytoreduction, **b** residual large abdominal wall defect and **c** abdominal wall reconstruction using biological mesh

The optimal approach for selected patients with peritoneal malignancy involves complete macroscopic tumour removal, known as cytoreductive surgery (CRS), combined with hyperthermic intraperitoneal chemotherapy (HIPEC)^1^, ^2^. The surgery generally involves a full midline laparotomy aiming for complete cytoreduction^3^, ^4^, ^5^, ^6^, often in patients who have had one or more abdominal procedures previously. Thus, at referral, many have an incisional hernia, and some have tumour infiltration of the abdominal wall (*Figs* 1 and 2), and require abdominal wall reconstruction after CRS and HIPEC.

Following laparotomy, reported rates of incisional hernia range from 9 to 20 per cent^7^, ^8^, ^9^, with most incisional hernias occurring within the first 5 years after laparotomy^10^, ^11^.

To achieve complete cytoreduction, multiple visceral resections as well as excision of large portions of the abdominal wall may be necessary (*Fig*. 2) and the majority of patients require bowel resections, either with reanastomosis or an abdominal wall stoma^12^. Several studies^13^, ^14^, ^15^, ^16^, ^17^ have demonstrated increased rates of wound complication in patients undergoing CRS and HIPEC (5–35 per cent) that may be related to prolonged operating time, hypothermia, bowel resection, the effects of chemotherapy agents or heat damage from HIPEC, which increases cellular death and induces apoptosis in tumour and normal tissue^18^, ^19^, ^20^.

Reconstruction of the abdominal wall in patients undergoing CRS and HIPEC can be particularly problematic for a number of reasons. The defects may be large, and the parietal peritoneum is removed at CRS (*Fig*. 2) such that it is not possible to place a mesh extraperitoneally protecting the bowel. In addition, major component separation techniques^21^, ^22^ are almost never possible owing to previous abdominal wall interventions, the presence of a stoma in many patients, and a relative contraindication to opening up large subcutaneous spaces in patients with a known high risk of wound infection.

For these reasons, in 2004 the senior author instituted the use of biological mesh for patients undergoing CRS and HIPEC who required abdominal wall reconstruction. There is little information on the long‐term durability of biological mesh, the effects of wound sepsis on mesh integrity, or possible risks of bowel erosion when a mesh is placed in direct contact with the bowel. Patients having CRS and HIPEC undergo annual CT, allowing an opportunity to assess a number of these factors over time. The aim of this study was to review the use of biological mesh to reconstruct the abdominal wall in patients undergoing CRS and HIPEC to establish immediate and long‐term outcomes.

## Methods

The study comprised an analysis of a dedicated peritoneal malignancy database, cross‐linked to a hospital registry of all biological mesh utilization. All patients who had abdominal wall reconstruction with biological mesh during CRS and HIPEC for pseudomyxoma peritonei (PMP) or colorectal peritoneal metastases (CPM) between 2004 and 2015 were identified.

The following information was retrieved for all patients: demographic data, diagnosis (PMP or CPM), primary or recurrent treatment, direct involvement of the abdominal wall or presence of an incisional hernia at presentation, operative details (duration, completeness of cytoreduction, type of HIPEC), mesh type and size. Completeness of cytoreduction was categorized as described by Jacquet and Sugarbaker^23^; patients with CC0 and CC1 were deemed to have undergone complete CRS.

All patients had standard preoperative mechanical bowel preparation, prophylactic antibiotics and venous thromboembolic prophylaxis. Abdominal access was via an elliptical midline laparotomy incision, from the xiphisternum to the symphysis pubis, excising the umbilicus and midline scars, incisional hernia or midline disease. Any disease involving areas of the abdominal wall lateral to the midline, for example in laterally placed port sites or lateral transverse incisions, was excised widely to achieve complete CRS. CRS involved various peritonectomies (parietal, pelvic and subdiaphragmatic) and visceral resections followed by HIPEC at 42 °C, using the open Colosseum technique as described previously^24^.

The abdominal wall was reconstructed after completion of HIPEC and after all anastomoses had been completed. Routine fascial closure was by continuous 1/0 nylon suture where tension‐free apposition was possible. Significant defects were closed using a biological mesh; the mesh was sutured with interrupted 2/0 Surgipro II™ sutures (Medtronic, Watford, UK) deep to the musculature of the abdominal wall (*Fig*. 2).

Patients were followed up according to standard PMP and CPM protocols, with annual CT of the abdomen and pelvis (without Valsalva manoeuvre). Definitive histology, short‐term surgical complications within 30 days of operation (wound infection, seroma, anastomotic leak, fistula to abdominal wall) and long‐term outcomes (hernia recurrence, disease recurrence on the abdominal wall or elsewhere, death) were recorded.

## Results

Thirty‐three patients (22 women) underwent CRS, HIPEC and abdominal wall reconstruction using biological mesh between 2004 and 2015. These 33 patients accounted for 2·7 per cent of 1229 patients treated by CRS and HIPEC over that interval. The mean age of the patients was 53·4 (range 19–82) years. Twenty‐three patients had PMP, of whom 19 had low‐grade disease. The other histological diagnoses were CPM in six and appendiceal adenocarcinoma in four patients.

Overall 18 of 33 patients had undergone CRS and HIPEC previously. The main cause of abdominal wall loss was direct abdominal wall involvement in 25 patients and an incisional hernia in the remaining eight. Mean total duration of surgery was 486 (range 120–795) min and the mean mesh size was 345 (range 50–654) cm^2^. The most frequently used biological mesh was Permacol™ (Medtronic) (27 patients) followed by EGIS™ (Raise Healthcare, Birmingham, UK) (4 patients) and Strattice™ (Allegran, Marlow, UK) (2 patients). The majority of patients had complete CRS (28); the remaining five underwent maximum tumour debulking, as outlined previously^25^.

**Figure 3:**
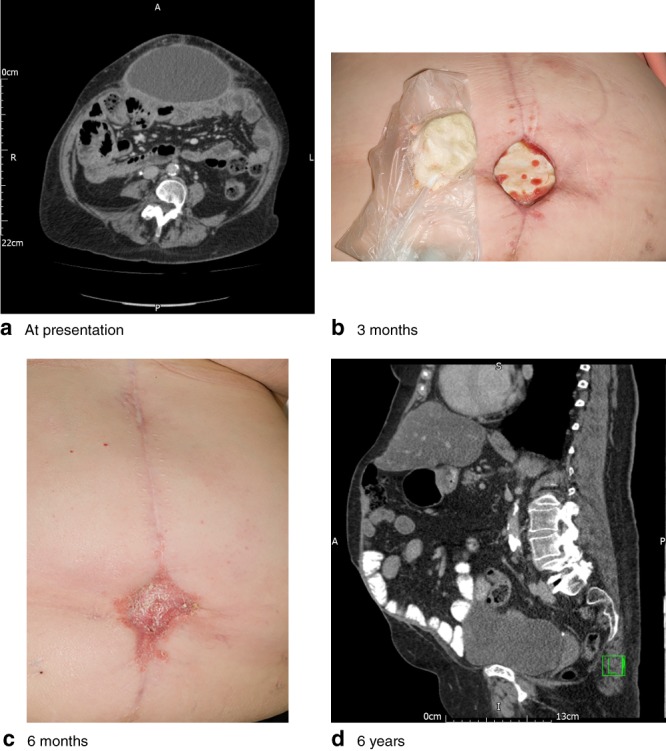
Images from a patient with an infected seroma that was debrided surgically: **a** at presentation, **b** 3 months, **c** 6 months and **d** 6 years after surgery

Overall, ten of 33 patients developed wound infection within the first 30 days after surgery. Six of these required antibiotic treatment, whereas the rest were treated with bedside drainage and regular wound dressing. Four patients had a seroma; one required seroma excision and vacuum‐assisted closure therapy application (*Fig*. 3). Two patients developed an enterocutaneous fistula in the early postoperative period; one was secondary to an anastomotic leak from a colorectal anastomosis and the other was a small bowel fistula, presumably from an undiagnosed intraoperative small bowel injury. Both fistulas settled with conservative treatment by control of sepsis and parenteral nutrition.

During a mean follow‐up of 48 (range 4–122) months, four of 33 patients died from progressive malignancy. A further 15 had disease recurrence or progression. In eight of these 15 patients, abdominal wall recurrence was documented and in seven this was associated with extensive intra‐abdominal disease. Isolated abdominal wall recurrence was noted in one of the 15 patients with recurrence. A further five of 33 had a CT‐documented incisional hernia at follow‐up. The hernia was symptomatic in one, and repair was offered but postponed at the patient's request. Two of the five hernias were detected within 1 year of abdominal wall reconstruction, one at 5 years and two at 10 years after the index procedure. All five hernias were at the edge of the mesh, two at the inferior edge and three at the lateral edge.

## Discussion

Prolonged surgery and large abdominal wall defects, combined with the adverse effects of HIPEC on wound healing, limit the available options for abdominal wall reconstruction after CRS and HIPEC. The high rate of wound complications in CRS and HIPEC (5–35 per cent)^13^, ^14^, ^15^, ^16^, ^17^ is a relative contraindication to anterior compartment separation as described by Ramirez and colleagues^21^. In addition, posterior component separation techniques^22^ are not achievable after major intra‐abdominal and abdominal wall surgery, including parietal peritonectomy. The use of synthetic or composite mesh is not advisable after CRS and HIPEC owing to the higher infection risk and loss of the peritoneum, such that the mesh cannot be placed in the extraperitoneal space to protect the bowel.

Introduced in the 1990s, biological meshes allow tissue infiltration and regeneration by attracting native fibroblasts and neovascularization^26^, ^27^. There are several products available in the market, Permacol™ being one of the first to be introduced into clinical practice. It is manufactured from porcine‐derived dermal sheets, mainly type 1 collagen. The manufacturing process involves removal of cellular material and treatment so that naturally occurring cross‐linking is encouraged. Strattice™ is also derived from acellular porcine dermis but is not cross‐linked^26^, ^27^, similar to EGIS™. Smart and co‐workers^27^ undertook a literature review and evaluated the performance of biological meshes in abdominal wall hernias. They reported that Permacol™ had the best outcomes overall, especially in challenging situations (high infection risk environment). With use of Permacol™, hernia recurrence rates were up to 15 per cent in the majority of studies, although one study^28^ reported rates of 41 per cent in patients with fistulas and/or laparostomies. In addition, Permacol™ had the longest time to failure, particularly in infected or contaminated wounds.

Patients in the present series mainly had clean or clean‐contaminated wounds but, as noted above, those undergoing CRS and HIPEC should be considered a high‐risk group for wound infection. The present findings reflect those of Smart *et al*.^27^ with a hernia recurrence rate of 15 per cent (5 of 33); however, follow‐up here was significantly longer than in any published series, either in patients with peritoneal malignancy or general surgical patients. In addition, three of the five hernia recurrences were detected 5 or 10 years later, well after the follow‐up period of the majority of studies reported in the literature.

In a series of eight patients undergoing CRS and HIPEC followed by abdominal wall reconstruction with a biological mesh, Boutros and colleagues^29^ reported one hernia recurrence during a much shorter follow‐up period of about 6 months. Nunez and co‐workers^17^ studied 213 consecutive patients undergoing CRS, HIPEC and abdominal wall reconstruction who had abdominal wall disease. Overall, only 10 per cent of the patients underwent abdominal wall reconstruction with a mesh, although the type of mesh (biological or synthetic) was not documented. Wound infection rates of up to 40·9 per cent in the mesh group were reported. In a similar German study, Struller *et al*.^30^ reported abdominal wall morbidity in 271 consecutive patients undergoing CRS and HIPEC for PMP, CPM or mesothelioma. Interestingly, no patient required abdominal wall reconstruction with mesh. The hernia rate was 7 per cent and full abdominal wound dehiscence occurred in 4 per cent during a follow‐up of 38 months, despite a relatively low wound infection rate of 14 per cent. The wound complication rate of 30 per cent in the present series falls within the reported rates in the literature, and it is therefore unlikely that the use of mesh contributes to wound infection. However, Nunez and colleagues^17^ noted that use of mesh (type not specified) significantly increased wound complications. In the present study, the two instances of early enterocutaneous fistula were not mesh‐related, and both patients were managed conservatively with percutaneous drainage, antibiotics and parenteral nutritional support.

This study has limitations in that the numbers were relatively small, and there was a lack of controls and randomization. Despite these limitations, important conclusions can be drawn. Abdominal wall reconstruction is challenging in patients with peritoneal malignancy, and the present results indicate that the use of biological mesh is a safe, reliable and robust way to reconstruct abdominal wall defects that are not amenable to tension‐free primary closure. Despite wound infections and intestinal fistulas, the biological mesh was effective and durable with an acceptable rate of postoperative hernia recurrence, most of which were asymptomatic.
